# Long COVID in France: prevalence, management and long-term impact: Tatjana Makovski

**DOI:** 10.1093/eurpub/ckac129.282

**Published:** 2022-10-25

**Authors:** TT Makovski, V Decio, L Carcaillon-Bentata, C Alleaume, N Beltzer, O Robineau, A Gallay, S Tebeka, J Coste

**Affiliations:** 1 Department of Non-communicable Diseases, Santé Publique France, Saint-Maurice, France; 2 Department of Alert and Crisis, Santé Publique France, Saint-Maurice, France; 3 Sorbonne Université, Inserm, Paris, France; 4 University Lille, Centre Hospitalier de Tourcoing, Tourcoing, France

## Abstract

Certain percentage of population experiences persistent symptoms months after an acute Covid-19 episode (Long-COVID), with a significant impact on daily-life. Few studies exist on its prevalence and its impact among the general population. The main objective of this survey was to estimate the prevalence of Long COVID among the general adult population in France. Secondary objectives were to evaluate Long COVID management and to assess impact of this clinical condition on quality of life and mental health. Cross-sectional study was performed in March-April 2022 using an online self-administered questionnaire. The sample was selected by the quota method from a panel of volunteers. Its representativeness was ensured by appropriate weighting. Three groups were described: Long-COVID, COVID without persistent symptoms, never COVID. Post COVID-19 condition as defined by the WHO was applied for prevalence estimation. The prevalence was calculated by age and sex. Health care consumption and impact of Long COVID on quality of life and mental health will be studied comparing the three groups, using weighted adjusted polytomic regressions. Here, we present preliminary findings on Long COVID prevalence. There were 27,537 respondents, 52% females, mean age (SD) 49 (±16.5). Confirmed or probable COVID-19 was reported by 33.9% of participants; of whom 85.1% had confirmed laboratory test. Majority (65.1%) had COVID-19 <3 months ago. Long COVID concerned 1,086 (4%) participants. Prevalence was higher for females 4.6% vs. 3.3% for males, and among younger population for both sex groups. Overall, prevalence of Long COVID by age group was: 18-34 (6%), 35-49 (4.7%), 50-64 (3.4%), ≥65 (1.8%). This is a first estimation of Long COVID prevalence among the French population. Representativeness of the sample should be interpreted with caution due to a sample based on volunteers’ response. Ongoing analyses will provide clearer understanding of the impact of Long COVID.

**Key messages:**

• This is a first estimation of Long COVID prevalence among the French population.

• There is a significant portion of the French population impacted by persisting or reoccurring symptoms defined by Long COVID; its impact and care management will be further evaluated.

